# Adaptive Multi-Rate Compression Effects on Vowel Analysis

**DOI:** 10.3389/fbioe.2015.00118

**Published:** 2015-08-20

**Authors:** David Ireland, Christina Knuepffer, Simon J. McBride

**Affiliations:** ^1^Computational Informatics, Australian e-Health Research Centre, CSIRO, Brisbane, QLD, Australia; ^2^Asia-Pacific Centre for Neuromodulation, UQ Centre for Clinical Research, University of Queensland, Brisbane, QLD, Australia; ^3^School of Information Technology and Electrical Engineering, University of Queensland, Brisbane, QLD, Australia

**Keywords:** speech compression, adaptive multi-rate codec, vowel sounds, speech processing

## Abstract

Signal processing on digitally sampled vowel sounds for the detection of pathological voices has been firmly established. This work examines compression artifacts on vowel speech samples that have been compressed using the adaptive multi-rate codec at various bit-rates. Whereas previous work has used the sensitivity of machine learning algorithm to test for accuracy, this work examines the changes in the extracted speech features themselves and thus report new findings on the usefulness of a particular feature. We believe this work will have potential impact for future research on remote monitoring as the identification and exclusion of an ill-defined speech feature that has been hitherto used, will ultimately increase the robustness of the system.

## 1 Introduction

Detection of a pathological voice from a digitally sampled waveform has long been established in a clinical environment; however, there is growing interest in capturing and using speech data-sets in a naturalistic environment. The ubiquitous use of smart-phone technology presents many open questions as to their efficacy in remote monitoring. The name smart-phone albeit common is rather ambiguous; they are in-fact a computer with a cellular transceiver and sensor array. Using a combination of the microphone, cellular transceiver, and Internet connectivity, gives some rather alluring possibility of obtaining large, naturalistic data-sets of speech acoustics that could be captured, post-processed and logged remotely. So-called remote or tele-monitoring is a rapidly growing field that aims to provide fast and frequent data collection in order to minimize the frequency of clinic visits and ultimately alleviate the workload of medical personnel. Specific examples for speech signals can be found in Little et al. (2009), Tsanas et al. (2010), and Arora et al. (2014). In most instances, specialized audio recording equipment was used. However, in gathering this data, it is likely that the signal would be compressed to allow for successful terrestrial communication and for storage. The adaptive multi-rate (AMR) codec is an audio compression format optimized for speech coding and widely used in the Global System for Mobile Communications standard (GSM). The AMR speech coder selects the rate adaptively depending on the channel condition. At present, AMR encoding comprises the narrow-band codec (AMR-NB), which encodes at 200–3400 Hz at variable bit ranges ranging from 4.75 to 12.2 kilobits per second (kbps), and the wideband codec (AWB-WB) which uses a bandwidth of 50–7000 Hz with bit-rates ranging from 6.6 to 23.85 kbps, achieving a higher quality of speech intelligibility. AMR-WB is now the default speech codec for the wideband code division multiple access (WCDMA) 3G systems.

Processing speech signals for detecting pathological biomarkers can be done in a variety of ways. The most common is the extraction of vowel sounds. Vowel sounds are produced when the vocal cords are resonating with the vocal tract open and fixed in position. In neurodegenerative diseases, such as Parkinson’s disease, there is preliminary evidence for abnormal vowel articulation even at the early stages of the disease (Sapir et al., 2007; Skodda et al., 2011, 2012). Healthy voices produce vowel sounds that are mostly periodic with a fundamental frequency *f*_0_ and uniform in amplitude; in contrast, pathological voices, show deviations in the fundamental frequency and amplitude of the articulated sound. Two common features to quantify this effect are *jitter* and *shimmer*. The first characterizes deviation in the fundamental frequency while the latter quantifies deviations in the amplitude. It is also commonplace to compute the formants of a vowel sound. Formants are the resonant frequencies of the vocal tract. If the vocal tract is fixed, formant computation can measure the placement and use of the speech articulators, which includes the lips, teeth, tongue, alveolar ridge, hard and soft palate, uvula, and glottis. A more modern feature is the mel-frequency cepstrum coefficients (MFCC). These coefficients are derived from a cepstral representation of the frequency spectrum of the audio signal. The spectrum is filtered according to a mel-scale which approximates the human auditory system response more closely than frequency bands spaced linearly across the spectrum. MFCC have shown promise in detecting pathological voices (Godino-Llorente et al., 2006). Although the use of these features have been shown useful in high-quality datasets, their efficacy on signals corrupted by compression is largely unknown. Thus, it is prudent to fully investigate the effects telecommunication compression would have on the analysis of voice signals so that suitable robust features are identified and error-prone features discarded.

Compression artifacts in speech samples were first examined in Besacier et al. (2001) and Gonzalez et al. (2003). A comprehensive comparison is given for control and pathological voices in Gonzalez et al. (2003), which examined the MP3 audio compression at bit-rates 32, 64, 98, and 128 kbps. It was found bit-rates >96 kbps preserved the relevant acoustic properties. A more recent effort is given in Tsanas et al. (2012). Here a realistic simulation of a cellular network was used to investigate the efficacy of obtaining speech samples via remote monitoring of a cellular network. The implemented simulator uses the AMR-NB codec with a fixed bit-rate of 12.2 kbps. A data-set of speech samples from people with Parkinson’s diseases was *piped* into the simulator. Subsequently, speech data at the end of the pipe was processed to extract 132 speech features that are used to predict the severity of the Parkinson’s disease that is known *a priori*. This work concluded that the performance degradation caused by the audio compression and simulated channel noise would unlikely prohibit predicting the severity of Parkinson’s disease. This article differs from the aforementioned work in the following regards:

1. Here we examine AMR-based codecs which are currently the state-of-art for speech compression.
2. All currently available bit-rates and modes are tested.
3. Rather than relying on a machine learning algorithm as in Tsanas et al. (2012) to test for accuracy, we examine the changes in the features themselves and thus report new findings on the usefulness of a particular feature.

**Figure 1:**
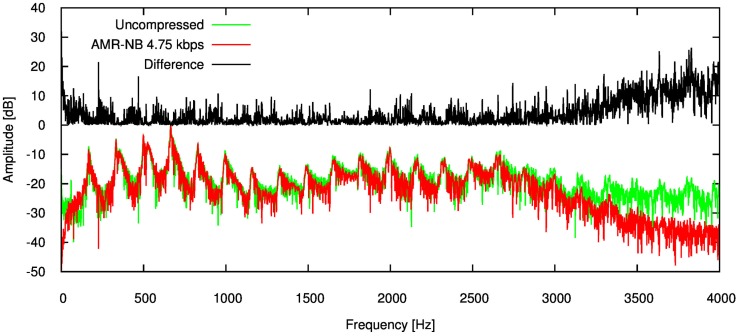
**Error for each speech feature when the audio signal is compressed using AMR-NB codec at 4.75 kbps**.

Anticipating the effects compression has on speech metrics is arduous. Figure 1 gives the power spectrum density (PSD) of an adult male speaker uttering a vowel. This Figure shows the PSD of the original signal, and after being compressed by the lowest possible bit-rate of the AMR codec (4.75 kbps). The difference between the two spectra is also given. Clearly the difference is large near the maximum limit of the frequency spectrum (>3000 Hz), which comprises the fine grain structure of the signal. However, there are differences across the spectrum likely caused by the codec encoding the signal using fewer bits than the original representation.

We believe that this work will have potential impact on future research on remote monitoring as the identification and exclusion of an ill-defined speech feature that has been hitherto used will ultimately increase the robustness of the system.

## 2 Materials and Methods

### 2.1 Speech corpus

The speech corpus used consisted of 45 men, 48 women, and 46 children (27 boys and 19 girls; age ranging from 10 to 12) and was first described by Hillenbrand et al. (1995) and subsequently released publicly at Hillenbrand (2008). The majority of the speakers (87%) were raised in Michigan, while the remainder was primarily from Illinois, Wisconsin, Minnesota, northern Ohio, and northern Indiana, all located in the United States of America. Audio recordings were made of subjects reading lists containing 12 vowels. Subjects read from one of 12 different randomizations of a list containing the words “*heed*”, “*hid*”, “*hayed*”, “*head*”, “*had*”, “*hod*”, “*hawed*”, “*hoed*”, “*hood*”, “*who’d*”, “*hud*”, “*heard*”, “*hoyed*”, “*hide*”, “*hewed*”, and “*how’d*”. A list of the extracted vowels in ASCII and the International Phonetic Alphabet library (IPA) is given in Table 1. Here, the average fundamental frequency and first and second formant frequencies are also given.

**Table 1:** **Average fundamental frequencies and formant frequencies of the vowel data-set produced by 45 men, 48 women, and 46 children**.

		**Vowel symbols**											
**IPA**		/i/	/I/	/e/	/ε/	/æ/	/*α*/	/ↄ/	/O/	/℧/	/u/	/Λ/	/з/
**ASCII**		*iy*	*ih*	*ey*	*eh*	*ae*	*ah*	*aw*	*oa*	*oo*	*uw*	*uh*	*er*

*F*_0_	M	243	192	267	189	278	267	283	265	192	237	188	263
	F	306	237	320	254	332	323	353	326	249	303	226	321
	C	297	248	314	235	322	311	319	310	247	278	234	307
*F*_1_	M	342	427	476	580	588	768	652	497	469	378	623	474
	F	437	483	536	731	669	936	781	555	519	459	753	523
	C	452	511	564	749	717	1002	803	597	568	494	749	586
*F_2_*	M	2322	2034	2089	1799	1952	1333	997	910	1122	997	1200	1379
	F	2761	2365	2530	2058	2349	1551	1136	1035	1225	1105	1426	1588
	C	3081	2552	2656	2267	2501	1688	1210	1137	149	1345	1546	1719

*All measurements in Hz*.

*M, males, F, females, C, children*.

The recordings were made with a digital audio recorder (Sony PCM-F1) and a dynamic microphone (Shure 570-S). Each obtained signal was low-pass filtered at 7.2 kHz, sampled at 16 kHz and quantized with 12-bits. The gain on an input amplifier was adjusted individually for each token so that the peak amplitude was at least 80% of the dynamic range of the analog to digital converter ensuring the amplitude peaks were not clipped. The reader is directed to Hillenbrand et al. (1995) for more information.

### 2.2 Speech compressing and analysis

In order to quantify the effects of the AMR compression, the voice samples are encoded and decoded using the OpenCORE library framework (OpenCore, 2013), which provides the required codecs. The speech signals are then analyzed using the open source, speech analysis software *Praat* (Boersma and Weenink, 2015) in their original and AMR compressed forms for the various bandwidths and bit-rates. In each instance the fundamental frequency, jitter, shimmer, harmonic-noise ratio (HNR), formant frequencies are obtained from *Praat*; the MFCCs are obtained from a program developed by the authors and released in open-source (Ireland, 2014). In this instance, the MFCC coefficients were produced from a set of 12 triangular filters spread between 50 and 4000 Hz and a fast Fourier transform size of 4096. All obtained values from Praat and the MFCC program are compared with and without compression.

## 3 Results

In order to quantify the error associated with the particular compression the following relative error equation was applied:

(1) $$E=\frac{M-M^{*}}{M}\times 100\%$$

where ℳ is a particular feature computed from uncompressed data, while ℳ* is the feature computed from speech signals that have undergone compression. If *E* > 0, then compression of the audio signal has caused the feature to be over-estimated, conversely if *E* < 0, the feature has been under-estimated.

To further support the error metric, tests of significance using Welch’s unequal variances t-test was used. This test is an adaptation of Student’s t-test, however it has been shown to be more reliable when two sample populations have unequal variances. The Bonferroni correction method is used to counteract the problem of multiple comparisons by adjusting the nominal test of significance (*α* = 0.05) based on the number of hypotheses resulting in a corrected threshold level denoted *α_c_*.

**Figure 2:**
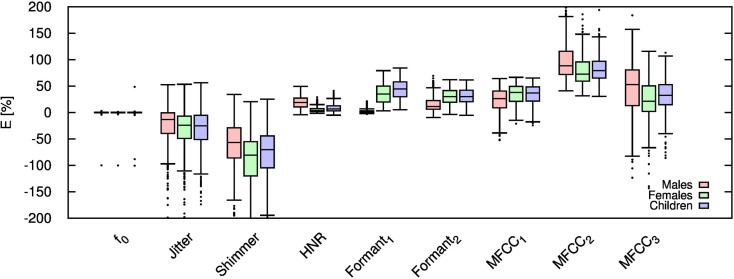
**Error for each speech feature when the audio signal is compressed using AMR-NB codec at 4.75 kbps**.

**Figure 3:**
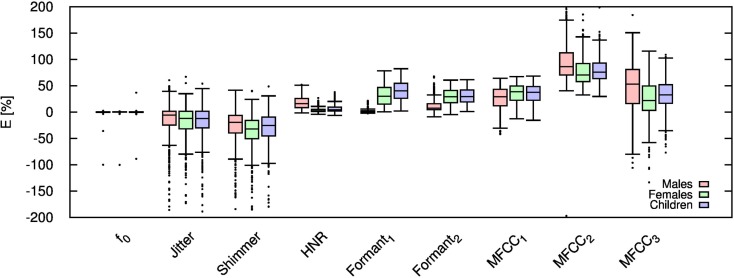
**Error for each speech feature when the audio signal is compressed using AMR-NB codec at 7.95 kbps**.

**Figure 4:**
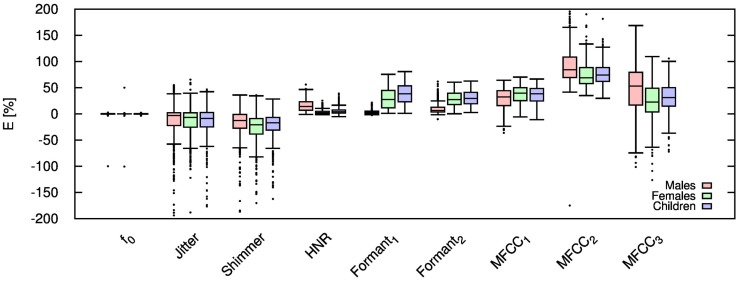
**Error for each speech feature when the audio signal is compressed using AMR-NB codec at 12.20 kbps**.

Table 2 shows the mean and SD of the resultant error when the audio is compressed using AMR-NB codec at all possible bit-rates. The complete data for bit-rates 4.75 kbps, 7.95 kbps, and 12.2 kbps are given in box-and-whisker form in Figures 2–4, respectively. The box-and-whisker plot was chosen because it readily displays key measures: the enclosed box depicts the lower quartile, median, and upper quartile while the arms extending from the box (whiskers) show the smallest and largest observation of the statistical data. Table elements in boldface represent the metrics that showed a high significance (p-value < *α_c_*).

**Table 2:** **Mean and SD (in brackets) of error for AMR-NB compression at various bit-rates**.

Feature	Gender	Bitrate (kbps)							
		4.75	5.15	5.90	6.70	7.40	7.95	10.2	12.2
*f*_0_	M	0 (4)	0 (4)	0 (2)	0 (4)	0 (0)	0 (5)	0 (4)	0 (4)
	F	0 (4)	0 (5)	0 (6)	0 (6)	0 (4)	0 (6)	**−**1 (7)	0 (5)
	C	0 (6)	**−**1 (9)	**−**1 (8)	**−**1 (7)	**−**1 (7)	0 (4)	**−**1 (7)	0 (0)
Jitter	M	**−24 (43)**	**−23 (42)**	**−23 (39)**	**−19 (42)**	**−18 (38)**	**−**16 (36)	**−**16 (38)	**−**18 (28)
	F	**−33 (40)**	**−30 (40)**	**−26 (35)**	**−23 (33)**	**−**19 (30)	**−**18 (31)	**−**16 (26)	**−**27 (31)
	C	**−33 (44)**	**−29 (40)**	**−27 (39)**	**−24 (37)**	**−22 (35)**	**−19 (34)**	**−17 (32)**	**−**22 (26)
Shimmer	M	**−68 (59)**	**−46 (48)**	**−42 (48)**	**−33 (39)**	**−29 (37)**	**−27 (35)**	**−25 (34)**	**−18 (28)**
	F	**−92 (59)**	**−57 (45)**	**−56 (48)**	**−43 (43)**	**−38 (40)**	**−38 (39)**	**−30 (31)**	**−27 (31)**
	C	**−80 (57)**	**−50 (43)**	**−48 (45)**	**−38 (40)**	**−34 (38)**	**−32 (37)**	**−27 (30)**	**−22 (26)**
HNR	M	**2 (3)**	**2 (3)**	**2 (3)**	**2 (3)**	**2 (3)**	**2 (3)**	**2 (3)**	**2 (3)**
	F	5 (5)	5 (5)	4 (4)	4 (4)	4 (4)	4 (4)	4 (4)	4 (4)
	C	9 (8)	8 (8)	7 (7)	7 (7)	7 (7)	7 (7)	6 (7)	6 (6)
*F*_1_	M	16 (14)	15 (14)	13 (12)	13 (13)	13 (13)	13 (12)	11 (11)	11 (11)
	F	**36 (18)**	**35 (18)**	**31 (19)**	**31 (19)**	**32 (19)**	**32 (19)**	**29 (19)**	**29 (19)**
	C	**44 (18)**	**43 (18)**	**40 (19)**	**40 (19)**	**40 (19)**	**40 (18)**	**39 (19)**	**38 (19)**
*F_2_*	M	**20 (11)**	**19 (11)**	**17 (11)**	**17 (11)**	**18 (11)**	**18 (11)**	**16 (11)**	**16 (10)**
	F	**30 (13)**	**30 (14)**	**29 (13)**	**29 (13)**	**29 (14)**	**29 (14)**	**28 (13)**	**28 (13)**
	C	**31 (13)**	**31 (13)**	**30 (14)**	**30 (14)**	**30 (14)**	**30 (14)**	**30 (13)**	**30 (14)**
MFCC_1_	M	**23 (22)**	**24 (22)**	**25 (22)**	**25 (21)**	**26 (21)**	**26 (21)**	**29 (20)**	**29 (19)**
	F	**35 (18)**	**35 (17)**	**35 (17)**	**35 (17)**	**35 (17)**	**36 (17)**	**37 (16)**	**37 (16)**
	C	**34 (18)**	**35 (17)**	**35 (17)**	**35 (17)**	**35 (17)**	**35 (17)**	**36 (16)**	**36 (16)**
MFCC_2_	M	**101 (48)**	**101 (48)**	**98 (47)**	**98 (47)**	**98 (47)**	**98 (47)**	**95 (42)**	**95 (42)**
	F	**83 (25)**	**83 (24)**	**81 (24)**	**81 (24)**	**80 (24)**	**80 (23)**	**78 (21)**	**78 (21)**
	C	**83 (25)**	**83 (24)**	**81 (24)**	**81 (24)**	**80 (24)**	**80 (23)**	**78 (21)**	**78 (21)**
MFCC_3_	M	**47 (51)**	**48 (51)**	**48 (51)**	**48 (51)**	**48 (51)**	**48 (50)**	**48 (49)**	**48 (48)**
	F	**24 (37)**	**25 (37)**	**26 (36)**	**26 (36)**	**25 (36)**	**25 (35)**	**24 (34)**	**25 (34)**
	C	**33 (31)**	**34 (30)**	**34 (30)**	**34 (30)**	**33 (30)**	**33 (30)**	**32 (29)**	**32 (29)**

*Table elements in boldface represent metrics that showed a significant difference compared to metrics based on uncompressed audio files*.

*Gender: M, males, F, females, C, children*.

Referring to Table 2, it is apparent that *f*_0_, and HNR showed very little distortion when compressed using AMR-NB; this was supported by the Welch t-test, which shows no significance at any bit-rate except for HNR in males. The MFCC, formants, jitter, and shimmer showed significant distortion with no noticeable improvement when the bit-rate increased. The Welch t-test shows the null-hypothesis is disproved across all bit-rates and gender groups for shimmer, MFCC, *F*_1_, and *F_2_*. Jitter at higher bit-rates (>7.4 kbps) showed no significance according to the Welch t-tests.

The jitter and shimmer errors indicate the estimated features are being consistently over-estimated for all bit-rates and genders. Conversely, the formants and MFCC are seen to be consistently under-estimated for all bit-rates and all genders. Except for the MFCC, male audio signals displayed the lowest errors followed by women and children.

**Figure 5:**
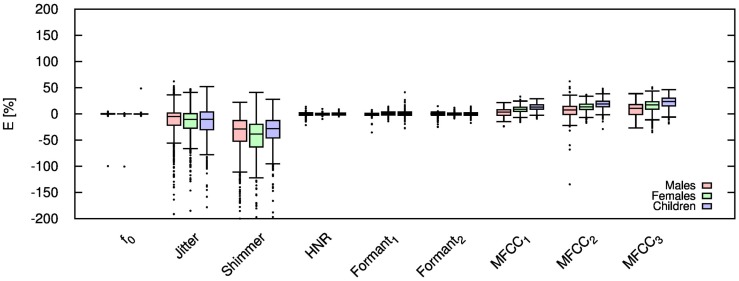
**Error for each speech feature when the audio signal is compressed using AMR-WB codec at 12.65 kbps**.

**Figure 6:**
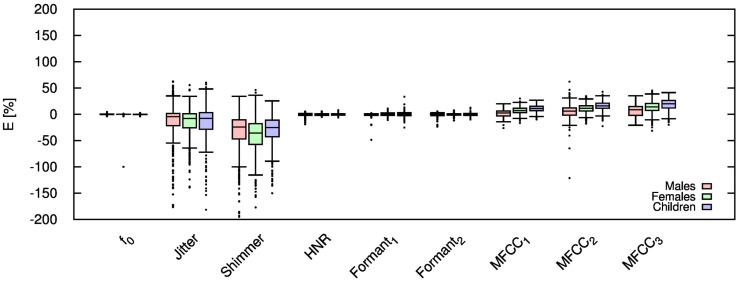
**Error for each speech feature when the audio signal is compressed using AMR-WB codec at 18.25 kbps**.

**Figure 7:**
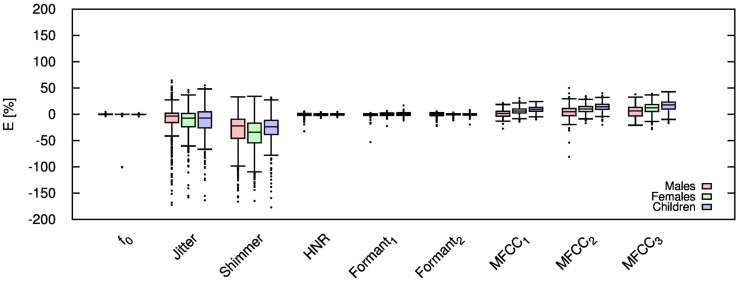
**Error for each speech feature when the audio signal is compressed using AMR-WB codec at 23.85 kbps**.

Table 3 shows the mean and SD (in brackets) of the resultant error when the audio is compressed using the AMR-WB codec at all possible bit-rates.Table elements in boldface represent metrics that show a high significance using the Welch t-test. The complete data for bit-rates 12.65 kbps, 18.25 kbps, and 23.85 kbps are given in box-and-whisker form in Figures 5–7, respectively. These figures reflect the lowest and highest possible bit-rate currently possible using AMR-WB codec. Referring to the Table 3, jitter and shimmer are shown to still exhibit significant distortion when the audio signal is compressed. The Welch-t test shows significance for each gender group and bit-rate for shimmer. The jitter metric showed no significance for bit-rates >8.85 kbps. The remaining features however showed a significant reduction in error particularly when the bit-rate increased. As in the AMR-NB, jitter and shimmer showed a tendency to be over-estimated while the MFCC were under-estimated. Clearly the AMR-WB codec is superior as expected due to the higher bit-rate and frequency bandwidth.

**Table 3:** **Mean and SD (in brackets) of error for AMR-WB compression at various bit-rates**.

Feature	Gender	Bitrate (kbps)								
		6.60	8.85	12.65	14.25	15.85	18.25	19.85	23.05	23.85
*f*_0_	M	0 (5)	0 (4)	0 (4)	0 (4)	0 (0)	0 (0)	0 (0)	0 (0)	0 (0)
	F	**−**1 (9)	**−**1 (7)	0 (4)	0 (6)	0 (6)	0 (4)	0 (6)	0 (6)	**−**1 (7)
	C	**−**1 (8)	**−**1 (7)	0 (2)	0 (4)	0 (4)	0 (1)	0 (4)	0 (4)	0 (0)
Jitter	M	**−27 (45)**	**−21 (39)**	**−**15 (35)	**−**13 (34)	**−**13 (33)	**−**14 (34)	**−**12 (34)	**−**12 (32)	**−**12 (34)
	F	**−37 (40)**	**−27 (34)**	**−**16 (27)	**−**16 (29)	**−**16 (26)	**−**15 (26)	**−**15 (25)	**−**15 (26)	**−**14 (26)
	C	**−38 (43)**	**−27 (38)**	**−**16 (33)	**−**14 (30)	**−**16 (31)	**−**15 (32)	**−**13 (28)	**−**15 (31)	**−**13 (28)
Shimmer	M	**−83 (64)**	**−66 (59)**	**−38 (40)**	**−38 (43)**	**−36 (42)**	**−35 (39)**	**−33 (38)**	**−33 (38)**	**−32 (36)**
	F	**−105 (63)**	**−82 (53)**	**−44 (36)**	**−41 (34)**	**−42 (34)**	**−40 (32)**	**−38 (32)**	**−37 (31)**	**−37 (31)**
	C	**−91 (63)**	**−69 (50)**	**−33 (30)**	**−31 (29)**	**−31 (29**)	**−30 (27)**	**−28 (28)**	**−27 (24)**	**−28 (29)**
HNR	M	0 (2)	0 (3)	**−**1 (2)	**−**1 (3)	**−**1 (2)	**−**1 (3)	**−**1 (2)	**−**1 (3)	**−**1 (3)
	F	**0 (2)**	**0 (2)**	0 (1)	0 (1)	0 (1)	0 (1)	0 (1)	0 (1)	0 (1)
	C	**0 (3)**	**0 (1)**	0 (1)	0 (1)	0 (1)	0 (1)	0 (1)	0 (1)	0 (1)
*F*_1_	M	1 (4)	1 (3)	0 (3)	0 (3)	0 (3)	0 (2)	0 (3)	0 (2)	0 (2)
	F	3 (5)	3 (4)	1 (2)	1 (2)	1 (2)	0 (2)	0 (2)	0 (1)	0 (2)
	C	**5 (9)**	**4 (8)**	1 (4)	1 (4)	1 (4)	0 (3)	0 (3)	0 (3)	0 (2)
*F_2_*	M	0 (4)	0 (3)	**−**1 (2)	**−**1 (2)	**−**1 (2)	**−**1 (2)	**−**1 (2)	**−**1 (2)	**−**1 (2)
	F	0 (4)	1 (3)	0 (2)	0 (2)	0 (1)	0 (1)	0 (1)	0 (1)	0 (1)
	C	**2 (6)**	**2 (5)**	0 (2)	0 (3)	0 (2)	0 (2)	0 (2)	0 (2)	0 (2)
MFCC_1_	M	**10 (9)**	**9 (8)**	3 (7)	2 (7)	2 (7)	2 (6)	2 (6)	1 (6)	1 (6)
	F	**17 (8)**	**16 (7)**	**9 (7)**	**8 (7)**	**8 (6)**	**7 (6)**	**7 (6)**	**6 (6)**	**6 (6)**
	C	**22 (8)**	**19 (7)**	**13 (6)**	**12 (6)**	**12 (6)**	**11 (6)**	**10 (6)**	**9 (6)**	**9 (6)**
MFCC_2_	M	12 (21)	10 (17)	7 (14)	6 (13)	6 (14)	5 (12)	5 (12)	5 (11)	5 (11)
	F	**20 (11)**	**17 (10)**	**13 (9)**	**12 (9)**	**12 (8)**	**11 (8)**	**11 (8)**	**10 (8)**	**10 (8)**
	C	26 **(12)**	**22 (10)**	**18 (9)**	**17 (9)**	**17 (9)**	**16 (8)**	**15 (8)**	**14 (8)**	**14 (8)**
MFCC_3_	M	12 (17)	9 (15)	8 (12)	8 (12)	7 (11)	7 (11)	6 (11)	5 (10)	5 (10)
	F	**21 (15)**	**19 (14)**	**16 (12)**	**15 (12)**	**14 (11)**	**13 (11)**	**13 (11)**	**11 (10)**	**12 (10)**
	C	**28 (14)**	**25 (13)**	**22 (11)**	**21 (11)**	**20 (11)**	**19 (11)**	**18 (10)**	**17 (10)**	**17 (10)**

*Table elements in boldface represent metrics that showed a significant difference compared to metrics based on uncompressed audio files*.

*Gender: M, males, F, females, C, children*.

### 3.1 Vowel analysis

**Figure 8:**
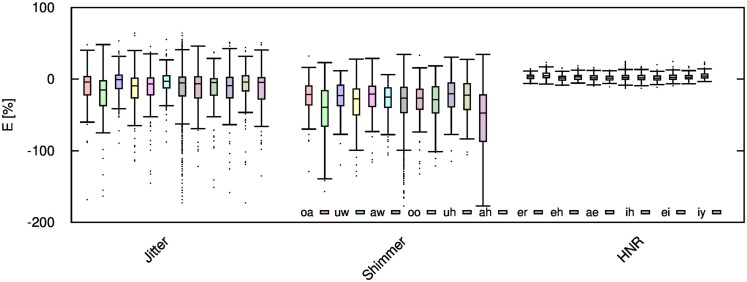
**Jitter, shimmer, and HNR errors for each spoken vowel when the audio signal is compressed using AMR-WB codec at 23.85 kbps**.

**Figure 9:**
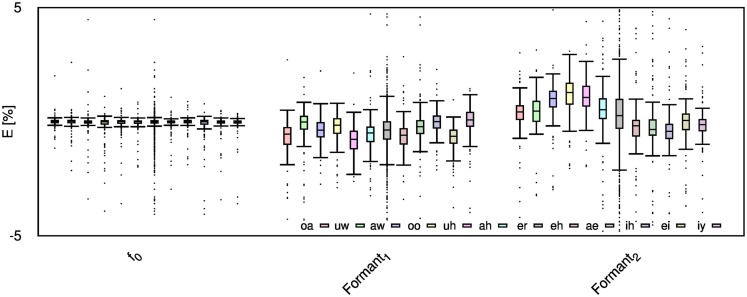
***f*_0_ and formant errors for each spoken vowel when the audio signal is compressed using AMR-WB codec at 23.85 kbps**.

**Figure 10:**
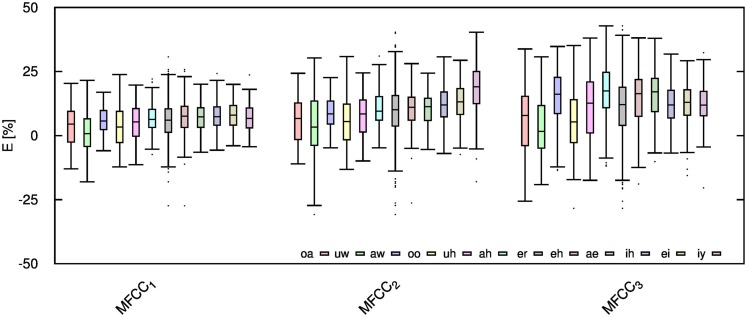
**MFCC errors for each spoken vowel when the audio signal is compressed using AMR-WB codec at 23.85 kbps**.

Given the significant distortion of some speech features, it is desirable to examine if these distortions are equal for each vowel, or if certain vowels are more sensitive to audio compression. To that end, the computed error values are further categorized into each unique vowel rather than gender. For brevity, this work only considers vowel signals compressed only with AMR-WB codec at 23.85 kbps; thus, this work reflects the highest obtainable accuracy with the AMR-WB codec. Figures 8–10 show the error for each vowel and feature. Here, the vowels are ordered based on the position of *F*_1_ in the frequency spectrum, where vowel *oa* has the lowest *F*_1_ and vowel *iy* has the highest; the remaining vowels are ordered in ascending order. Initially, it was suspected that this order shows a steady increase in error but Figures 8–10 show this not to be entirely true. Table 4 gives the order of the vowels with ascending mean and SD.

**Table 4:** **Vowel error in ascending order (left to right) sorted by mean and SD (in brackets)**.

Feature	Vowel error in ascending order											
*f*_0_	*oa*(*oa*)	*uw*(*ae*)	*ae*(*ah*)	*uh*(*uw*)	*ah*(*eh*)	*eh*(*iy*)	*iy*(*uh*)	*ih*(*ih*)	*er*(*er*)	aw(aw)	*ei(*ei*)*	*oo*(*oo*)
Jitt*er*	aw(*ah*)	*ah*(aw)	*ei*(*oa*)	*oa*(*ae*)	*er*(*iy*)	*ae*(*er*)	*iy*(*ei*)	*uh*(*uh*)	*eh*(*ih*)	*ih*(*uw*)	*oo*(*oo*)	*uw*(*eh*)
Shimm*er*	*ih*(aw)	aw(*oa*)	*oa*(*ah*)	*ei*(*uh*)	*uh*(*ih*)	*ah*(*ei*)	*eh*(*eh*)	*oo*(*ae*)	*er*(*oo*)	*ae*(*er*)	*uw*(*uw*)	*iy*(*iy*)
HNR	*ih*(*ih*)	*ae*(*ei*)	*oo*(aw)	*uw*(*uh*)	aw(*oa*)	*er*(*oo*)	*ah*(*ah*)	*iy*(*ae*)	*eh*(*eh*)	*oa*(iw)	*ei*(er)	*uh*(*iy*)
*F*_1_	*ae*(aw)	*oa*(*uh*)	*er*(*ah*)	*ei*(*oa*)	*iy*(*iy*)	*uw*(*ih*)	*eh*(*er*)	*ah*(*eh*)	*ih*(*oo*)	*oo*(*ei*)	*aw*(*ae*)	*uh*(*uw*)
*F_2_*	aw(*oa*)	*uh*(*ei*)	*ah*(*uh*)	*ae*(*iy*)	*oo*(aw)	*ei*(*ih*)	*er*(*oo*)	*eh*(*eh*)	*iy*(*ae*)	*ih*(*er*)	*oa(*ah*)*	*uw*(*uw*)
MFCC_1_	*uw*(*ae*)	*oo*(*iy*)	*oa*(aw)	*uh*(*ei*)	*er*(*ah*)	aw(*ih*)	*ah*(*er*)	*iy*(*oa*)	*ae*(*eh*)	*eh*(uh)	*ih*(*uw*)	*ei*(*oo*)
MFCC_2_	*uw*(*ae*)	*oo*(aw)	*oa*(*ah*)	*uh*(*ih*)	aw(*ei*)	*er*(*eh*)	*eh*(*uh*)	*ae*(*oa*)	*ah*(*oo*)	*ih*(her)	*ei*(*iy*)	*iy*(*uw*)
MFCC_3_	*uw*(*iy*)	*oo*(*ih*)	*oa*(*ei*)	*er*(*ae*)	*uh*(*eh*)	*ih*(*ah*)	*ei*(aw)	*iy*(*er*)	*aw*(*uw*)	*eh*(*oo*)	*ae*(*oa*)	*ah*(*uh*)

## 4 Discussion

An analysis of the effects of AMR-NB compression showed *f*_0_ and HNR to be almost unaffected by compression in any bit-rate or bandwidth for all genders. The HNR feature did show a consistent albeit small tendency to be over-estimated by as much as 9% for children. The formant frequencies and MFCC were found to be significantly over-estimated in the AMR-NB codec in any bit-rate while the jitter and shimmer values were found to undergo significant distortion by consistently being under-estimated by as much as 101%. Clearly, *f*_0_ and HNR from the given list of features are the only viable ones when using the AMR-NB codec. When comparing genders, it is apparent that males generally produce less error compared to females and children. This is likely due to the lower voice pitch inherent in male voices and thus most of the speech energy is lower in the spectrum. Error analysis when using AMR-WB codec showed *f*_0_, HNR, and the formant frequencies to be almost unaffected. The latter has shown significant improvement even at a bit-rate 6.60 kbps suggesting the increase in frequency bandwidth in AMR-WB allows the formant estimation algorithm in *Praat* to be more accurate. MFCC estimations have improved by no more than 26% at the lowest bit-rate for children. The error decreases as the bit-rate increases. The shimmer and jitter values still remained significantly over-estimated by as much as 105% and do not improve significantly as the bit-rate increases. It can thus be concluded that the reliance on the use of jitter and shimmer in remote monitoring using cellular data-sets must be entirely avoided. The use of MFCC and formant frequencies must be used with caution, particularly when the cellular system is only using the AMR-NB codec, such as the 2G network.

## Conflict of Interest Statement

The authors declare that the research was conducted in the absence of any commercial or financial relationships that could be construed as a potential conflict of interest.
